# ROS‐derived lipid peroxidation is prevented in barley leaves during senescence

**DOI:** 10.1111/ppl.13769

**Published:** 2022-09-12

**Authors:** Ginga Shimakawa, Anja Krieger‐Liszkay, Thomas Roach

**Affiliations:** ^1^ Department of Bioscience, School of Biological and Environmental Sciences Kwansei‐Gakuin University Sanda Japan; ^2^ Institute for Integrative Biology of the Cell (I2BC), CEA, CNRS Université Paris‐Saclay Gif‐sur‐Yvette France; ^3^ Department of Botany University of Innsbruck Innsbruck Austria

## Abstract

Senescence in plants enables resource recycling from senescent leaves to sink organs. Under stress, increased production of reactive oxygen species (ROS) and associated signalling activates senescence. However, senescence is not always associated with stress since it has a prominent role in plant development, in which the role of ROS signalling is less clear. To address this, we investigated lipid metabolism and patterns of lipid peroxidation related to signalling during sequential senescence in first‐emerging barley leaves grown under natural light conditions. Leaf fatty acid compositions were dominated by linolenic acid (75% of total), the major polyunsaturated fatty acid (PUFA) in galactolipids of thylakoid membranes, known to be highly sensitive to peroxidation. Lipid catabolism during senescence, including increased lipoxygenase activity, led to decreased levels of PUFA and increased levels of short‐chain saturated fatty acids. When normalised to leaf area, only concentrations of hexanal, a product from the 13‐lipoxygenase pathway, increased early upon senescence, whereas reactive electrophile species (RES) from ROS‐associated lipid peroxidation, such as 4‐hydroxynonenal, 4‐hydroxyhexenal and acrolein, as well as β‐cyclocitral derived from oxidation of β‐carotene, decreased. However, relative to total chlorophyll, amounts of most RES increased at late‐senescence stages, alongside increased levels of α‐tocopherol, zeaxanthin and non‐photochemical quenching, an energy dissipative pathway that prevents ROS production. Overall, our results indicate that lipid peroxidation derived from enzymatic oxidation occurs early during senescence in first barley leaves, while ROS‐derived lipid peroxidation associates weaker with senescence.

## 1 INTRODUCTION

Leaf senescence is an important nutrient‐recycling process in higher plants, and seen as the last stage of leaf development (Lim et al., 2007). It is endogenously regulated, but can be induced by exogenous parameters, such as stress. Endogenous regulation occurs via phytohormone and sugar levels, protein phosphorylation and potentially reactive oxygen species (ROS), which each have varying influence depending on the age of the leaf and the developmental state of the plant. During senescence, cellular constituents are degraded in a coordinated manner to enable nutrient availability for sink organs, such as seeds or new leaves, depending upon the developmental state of the plant and its growth strategy (i.e. monocarpic or perennial). Developmental senescence can be divided into two stages; first, ‘sequential senescence’ whereby resources are reallocated from old to young leaves, and second, ‘reproductive senescence’ of monocarpic species (e.g. annuals) in which resources are made available for seed production (Thomas, 2013). Leaves that house photosynthetic machinery are resources for growth, such as N‐rich light‐harvesting complexes (LHC), also called ‘antenna’, and the highly abundant RuBisCO enzyme.

ROS are clear candidates for chloroplast‐derived signals that may play a role in triggering senescence (Krieger‐Liszkay et al., 2019). Regarding developmental senescence, hydrogen peroxide (H_2_O_2_) has emerged as such a signal in mature monocarpic annual plants before flowering (Bieker et al., 2012; Zentgraf et al., 2022). In support of this hypothesis, several senescence‐associated transcription factors are responsive to H_2_O_2_ (Balazadeh et al., 2011; Garapati et al., 2015; Zentgraf & Doll, 2019). Furthermore, ROS‐mediated oxidative modifications (e.g. protein carbonyls, aldehydes/alkenals from lipid peroxidation, etc.) accumulate in senescing leaf chloroplasts during reproductive senescence, and there are clear overlaps between photo‐oxidative responses and senescence, although most of these reports are from plants under stress (Pintó‐Marijuan & Munné‐Bosch, 2014). Of note, H_2_O_2_ is produced from various peroxisome‐located oxidases in catabolism, as well as from dismutation of superoxide (O_2_^•−^) produced by electron leakage from electron transport chains of respiration and photosynthesis (Halliwell, 2006). The ROS singlet oxygen (^1^O_2_) is predominantly produced by excited chlorophyll in its triplet state in the photosystem II (PSII) reaction centre, or LHC in the PSII grana core (Dogra & Kim, 2020; Krieger‐Liszkay, 2005). ^1^O_2_ is highly oxidising, and has a prominent role in oxidative modifications of thylakoid membranes, leading to lipid peroxidation (Biswas & Mano, 2021; Triantaphylidès et al., 2008), and release of reactive electrophile species (RES) that can activate a large proportion of the ^1^O_2_ signalling pathway (Mano et al., 2019; Roach et al., 2018). Furthermore, oxidation of the β‐carotene in the PSII reaction centre releases the RES β‐cyclocitral that can be involved in ^1^O_2_‐mediated signalling of *Arabidopsis thaliana* (Ramel et al., 2013). Lipid oxidation via the lipoxygenases (LOX) pathway also leads to the production of six‐carbon aldehydes, such as hexanal (hexaldehyde) and oxylipins phytohormones called jasmonates. Besides functioning in abiotic and biotic stress responses, jasmonates may play a role in senescence signalling (Seltmann et al., 2010), particularly in monocot plants (Bachmann et al., 2002), as well as in the breakdown of the plastid envelope (Springer et al., 2016). However, if aldehyde/RES initiate signalling pathways that activate sequential leaf senescence in non‐stressed leaves is less clear.

In this study, we used two barley cultivars, cultivar Lomerit (*Hordeum vulgare* L., cv. Lomerit) and cultivar Carina (*Hordeum vulgare* L., cv. Carina), to investigate patterns of lipid peroxidation, as markers of ROS and LOX activity, during senescence of first leaves grown in a glasshouse under natural light regimes. In monocarpic annual crops, such as barley, sequential senescence occurs early in plant development to facilitate the growth of the shoot and new leaves (Distelfeld et al., 2014; Gregersen et al., 2008). Under outdoor conditions, the first leaf of barley cv. Lomerit and cv. Carina, when grown in small pots, start to senesce 2 weeks after sowing seeds (Shimakawa et al., 2020). In the first barley leaves of these cultivars, no differences in production rates of O_2_^•−^/H_2_O_2_, between early (17 days after sowing) and later (31 days after sowing) stages of senescence were found, while rates of ^1^O_2_ production increased in cv. Lomerit, but not in cv. Carina at the later stage (Shimakawa et al., 2020). Therefore, the question arises whether ROS/RES play a role in activating sequential senescence in non‐stressed plants. Considering profiles of ROS production in leaves of these cultivars during senescence (Krieger‐Liszkay et al., 2015; Shimakawa et al., 2020), we hypothesised that mechanisms are activated that protect against ROS production in the chloroplast, or prevent ROS‐mediated lipid peroxidation from propagating (i.e. antioxidants).

## 2 MATERIAL AND METHODS

### 2.1 Plant material

The winter barley cv. Lomerit and the spring cv. Carina were grown in pots (five to nine plants per 6 × 6‐cm pot) in commercial germination soil (Fruhstorfer Erde, Hawita), in non‐controlled conditions in summer (from June to July) in a greenhouse at the Institute of Botany, University of Innsbruck, Austria. Air temperature and light intensity immediately next to the plants (Figure S1) were measured with an Easy EL‐USB‐1‐LCD data logger (Lascar) and SE‐SQ520 sensor (Apogee Electronics), respectively. The first emerging unfolded leaf from the coleoptile, hereafter ‘first leaf’, of cv. Lomerit and cv. Carina were used for all experiments before and after onset of natural senescence (Figure S2).

### 2.2 Chlorophyll fluorescence and near‐infrared absorbance analyses

Maximum quantum efficiency of PSII photochemistry (*F*_v_/*F*_m_) and total photo‐oxidizable P700 (*P*_m_) were non‐invasively determined on attached leaves with a Dual‐PAM‐100 (Walz). For the chlorophyll fluorescence measurements, the first barley leaves were dark adapted for 15 min. A pulse‐amplitude modulated red measuring light (620 nm, 0.1 μmol photons m^−2^ s^−1^) was used to obtain *F*_o_ (minimum fluorescence from dark‐adapted leaves). For the determination of *F*_m_ (maximum fluorescence from dark‐adapted leaves), a short‐saturation flash (8000 μmol photons m^−2^ s^−1^, 300 ms) was provided by the LED array. *F*_v_/*F*_m_ was calculated as (*F*_m_ – *F*_o_)/*F*_m_ (Baker, 2008). For P700 measurements, the leaves were taken from the light. The transmittance of oxidised P700 (P700^+^) was measured by pulse‐amplitude modulated near‐infrared measuring lights (830 and 870 nm) (Klughammer & Schreiber, 1994). The light guide was restricted to minimum leaf width using black insulation tape (in total 0.45 cm^2^). The full oxidation level of P700 was determined by the 300‐ms short‐saturation flash after 10 s far‐red light illumination (730 nm), and the full reduction level of P700 was defined in the dark, resulting in the *P*_m_ values.

Non‐photochemical quenching (NPQ) of first leaves was measured with an Imaging‐PAM (Walz). Pulse‐modulated excitation, actinic illumination, and saturation flashes were achieved with a blue LED lamp with a peak emission of 450 nm. The actinic light intensity was set at 1250 μmol photons m^−2^ s^−1^, and NPQ was calculated as (*F*_m_ – *F*_m_′)/*F*_md_′, with *F*_m_′ the maximum fluorescence emission from light‐adapted leaves. *F*_m_ and *F*_m_′ were determined using saturating flashes (8000 μmol photons m^−2^ s^−1^).

### 2.3 Pigments, aldehydes and RES quantification

Plants were sampled in the afternoon and taken from direct sunlight (intensity 1000–1100 μmol photons m^−2^ s^−1^). Measurements of *P*_m_ were made of leaf segments without dark adaption. After cutting the leaf segments from the first leaf, the dimensions (width, length) were rapidly measured before freezing in liquid nitrogen in a 2 ml reaction tube with two 3‐mm glass beads. The process was repeated for each leaf segment one by one. Extraction was made in 1.0 ml acetonitrile by shaking at 30 Hz for 2 min. After centrifugation for 10 min at 29,000*g* and 4°C, the supernatant was split. An aliquot of the sample (15 μl injection volume) was used for simultaneous measurements of pigments, measured via a diode array detector (absorption at 440 nm), and tocopherols, measured with a fluorescence detector (Ex: 295 nm; Em: 325 nm), using an Agilent 1100 HPLC (Roach et al., 2017). Aldehydes and RES in 400 μl of the supernatant were derivatised with 2,4‐dinitrophenylhydrazine, along with 0.5 μM 2‐ethylhexanal (as internal standard) and 0.005% (wt/vol) of butylated hydroxytoluene, using LC–MS/MS (Roach et al., 2017).

### 2.4 Lipids and lipoxygenase activity

Plants were sampled for the other biochemical analyses, except for the freeze‐dried leaf segments. Total fatty acids (e.g. membranes, storage lipids, free fatty acids) of cv. Carina were extracted in 1.0 ml of methanol:toluene:sulphuric acid 40:12:1 (vol:vol:vol), containing 0.01% (wt:vol) butylated hydroxytoluene, and heptadecanoic acid (C17:0), as internal standard. After derivatisation to fatty acid methyl esters (FAMEs), samples were analysed by gas chromatography coupled with mass spectrometry (GC–MS), as previously described (Schausberger et al., 2019), and normalised to dry weight (DW). Relative changes were based on peak area for selected fragments of each fatty acid, whereas relative fatty acid composition after treatments were evaluated by peak areas of total ion current (TIC) chromatograms for each fatty acid. For thin‐layer chromatography of membrane lipids, lipids were extracted in chloroform/methanol (1:2, vol/vol), with extraction volume adjusted according to sample DW, before separation and detection on silica gel 60 plates with concentration zones (Merck Millipore) impregnated with ammonium sulphate, according to Wewer et al. (2013). Bands were assigned with pure standards purchased from Avanti Polar Lipids, and quantified using densitometry.

For measuring lipoxygenase activity, ground leaf segments of cv. Carina were extracted in 500 μl of 50 mM TRIS buffer, pH 7.0, centrifuged for 10 min at 29,000*g* and 4°C and 50 μl of the supernatant was added to 150 μl 50 mM TRIS buffer, pH 7.0, containing 2 μl of 5 mM linoleic acid dissolved in dimethyl sulfoxide in a UV‐transparent 96‐well plate (Brand). Lipid peroxide formation was measured at 234 nm every 15 s between 1 and 5 min at 30°C, with two technical replicates per biological replicate, using the 0.1 m^2^ mol^−1^ extinction coefficient of 27,000 and a 0.5 cm light path‐length. The activity was normalised to protein amount, measured in two technical replicates for each 5 μl extract, using the Bradford Assay (BioRad).

### 2.5 Spin‐trapping electron paramagnetic resonance measurements

Electron paramagnetic resonance (EPR) spin‐trapping assays for determining H_2_O_2_ were carried out using the spin trap 4‐pyridyl‐1‐oxide‐N‐tert‐butylnitrone (4‐POBN) (Sigma‐Aldrich). Leaves were vacuum‐infiltrated with a buffer (25 mM HEPES, pH 7.5, 5 mM MgCl_2_, 0.3 M sorbitol) containing the spin trap reagents (50 mM 4‐POBN, 4% ethanol, 50 μM Fe‐EDTA). Infiltrated leaves were placed into the buffer containing the spin trap reagents and illuminated for 1 h with white light (200 μmol photons m^−2^ s^−1^). At the end of the illumination time, the leaves were removed and the EPR signal of the solution was monitored. EPR spectra were recorded at room temperature in a standard quartz flat cell using an ESP‐300 X‐band (9.73 GHz) spectrometer (Bruker). The following parameters were used: microwave frequency 9.73 GHz, modulation frequency 100 kHz, modulation amplitude: 1 G, microwave power: 6.3 mW, receiver gain: 2 × 104, time constant: 40.96 ms; number of scans: 4. The EPR signal sizes were normalised by the total chlorophyll contents of each sample.

### 2.6 Statistics

Six random plants of each cultivar were selected at each of the six‐time points for measurements. For regression analysis of *P*_m_ with biochemical parameters, log‐normal fitting was conducted in OriginPro 2017G for both cultivars collectively, or with photosynthetic parameters for each cultivar separately. For calculating *p* values over time for both cultivars, or between cultivars over all time points, a univariate general linear model was used in IBM SPSS Statistics 25.

## 3 RESULTS

### 3.1 Leaf senescence and photosynthesis

Barely seeds of two cultivars, Carina and Lomerit, were sown on six occasions during two time courses, so that each of the two time courses ended with first leaves of differing degrees of senescence (Figure S1). This was chosen so leaves of different ages had experienced the same environmental conditions before measurements. First leaves were measured at 13, 20 and 27 days (time course 1), or 18, 25 and 32 days (time course 2), since seeds had been sown (Figure S1A). Light intensity in the naturally lit glasshouse used for growing plants reached 1300 μmol photons m^−2^ s^−1^ on cloudless days, and up to 250 μmol photons m^−2^ s^−1^ when overcast (Figure S1A). Due to partial shading from trees above, plants were subjected to fluctuating, rather than slowly changing, light intensities (Figure S1B). The average temperature during the entire growing period was 24.3°C, with typically a 10–15°C difference between maximum day and minimum night temperatures (Figure S1A). Notably, plants from the first course had experienced warmer maximum temperatures in the week before measurement than those from the second time course (Figure S1).

**FIGURE 1:**
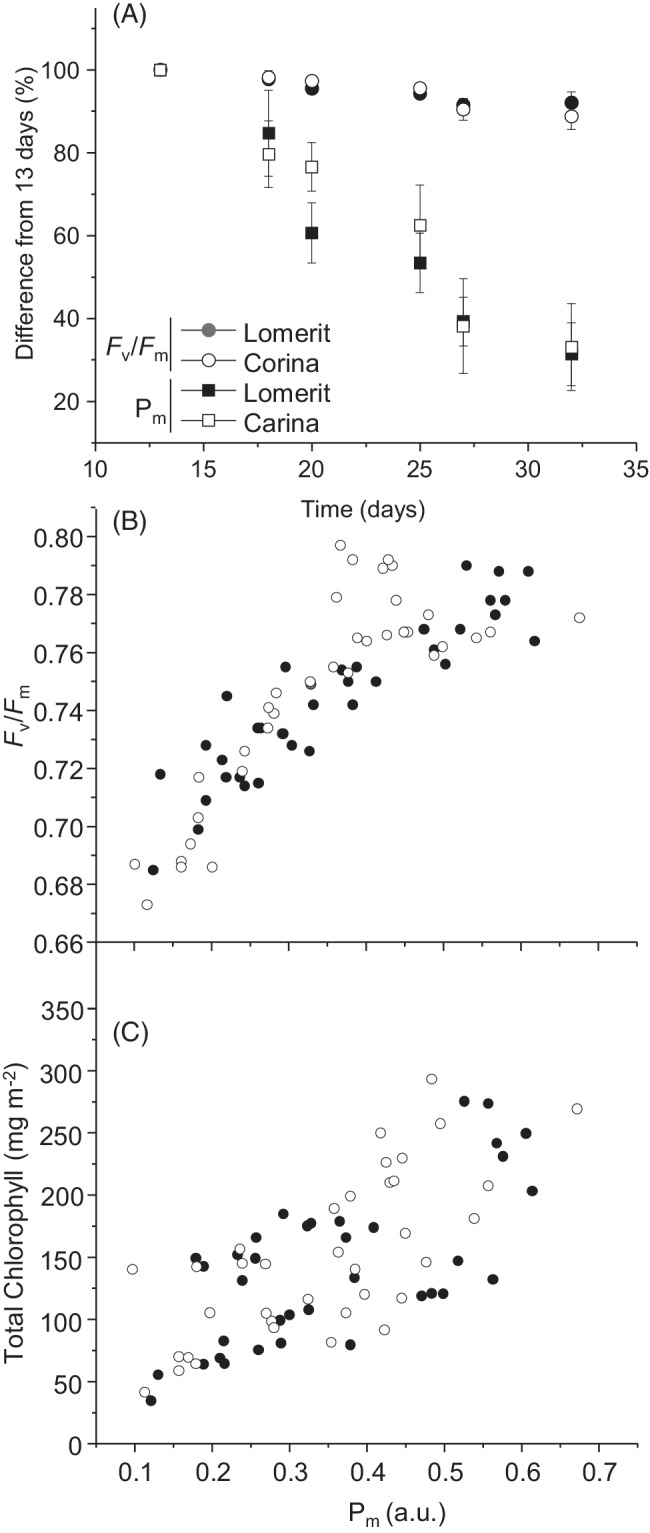
Changes in photosynthesis‐related parameters during sequential senescence in barley leaves of cv. Lomerit (closed symbols) and cv. Carina (open symbols). (A) Maximum quantum yields of photosystem II (*F*_v_/*F*_m_; circles) and maximum redox change of photosystem I (*P*_m_; squares) of first‐emerging leaves after 13, 20 and 27 days (first time course) or 18, 25 and 32 days (second time course) after sowing seeds, with all changes shown relative to values of pre‐senescent leaves (13 days), means ± sd, *n* = 6 for each genotype at each time point. (B, C) Relationship of *P*_m_ values with (B) *F*_v_/*F*_m_, and (C) total chlorophyll (*a* + *b*) concentrations, with each data point representing an individual leaf

As senescence indicators, we measured maximum quantum efficiency of PSII (*F*_v_/*F*_m_), maximum photo‐oxidizable P700 levels (*P*_m_), and total chlorophyll content. In accordance with a previous study of the same cultivars (Shimakawa et al., 2020), the magnitude of decline of *P*_m_ during senescence was greater than the loss of *F*_v_/*F*_m_ (Figure 1A), and *P*_m_ declined earlier than *F*_v_/*F*_m_ (Figure 1B). Thus, we chose *P*_m_ as a reference to the degree of leaf senescence throughout this study. This non‐destructive measurement was made immediately before biochemical measurements, enabling correlation of *P*_m_ with other parameters for 72 individual first leaves, with six replicate leaves for each cultivar for each of the six different ages of first leaves. Levels of total chlorophyll linearly correlate with *P*_m_, with Pearson *R* values of 0.67 and 0.78 for cv. Lomerit and cv. Carina, respectively, (Figure 1C). In all three senescent indicators, no distinguishable difference between cultivars is observable (Figure 1).

**FIGURE 2:**
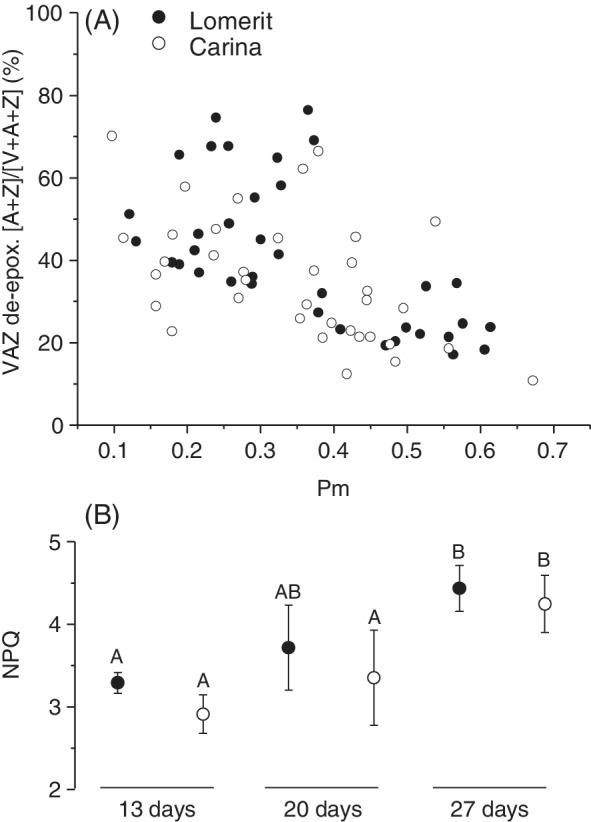
Relationship between maximum redox change of photosystem I (*P*_m_), used as a senescence marker, and xanthophyll cycle de‐epoxidation ratios (VAZ de‐epox.), and changes in non‐photochemical quenching (NPQ), during sequential senescence of first‐emerging barley leaves of cv. Lomerit (closed symbols) and cv. Carina (open symbols). (A) Each data point represents an individual leaf, V: Violaxanthin; A, Antheraxanthin; Z, zeaxanthin. (B) NPQ values of leaves after 13, 20 and 27 days after sowing seeds, means of each time point ±sd, with different letters denoting significant differences at *p* < 0.05, *n* = 6 leaves for each genotype at each time point

Regarding photosynthesis‐related carotenoids, levels of lutein and neoxanthin remained similar relative to total chlorophyll, while antheraxanthin and zeaxanthin levels increased and violaxanthin decreased (Figure S3; Table S1), significantly shifting the xanthophyll de‐epoxidation ratio at later stages of senescence (Figure 2A; Table S1), also when chlorophyll content was used as a senescence marker (Figure S4). Activation of the xanthophyll cycle agrees with the significant increase in NPQ observed in late‐senescent leaves (Figure 2B), i.e. when *P*_m_ values had decreased by ca. 60% (Figure 1A). During senescence, levels of β‐carotene found in the reaction centre of PSII and the core complexes of PSI decreased relative to total chlorophyll (Figure S3; Table S1), showing overall that changes in pigments are not uniform and that reaction centres are likely broken down before antenna. In support of this, the chlorophyll *a*:*b* ratio significantly decreased as senescence progressed (Figure S4; Table S1).

### 3.2 Reactive electrophile species, hexaldehyde and lipoxygenase activity

**FIGURE 3:**
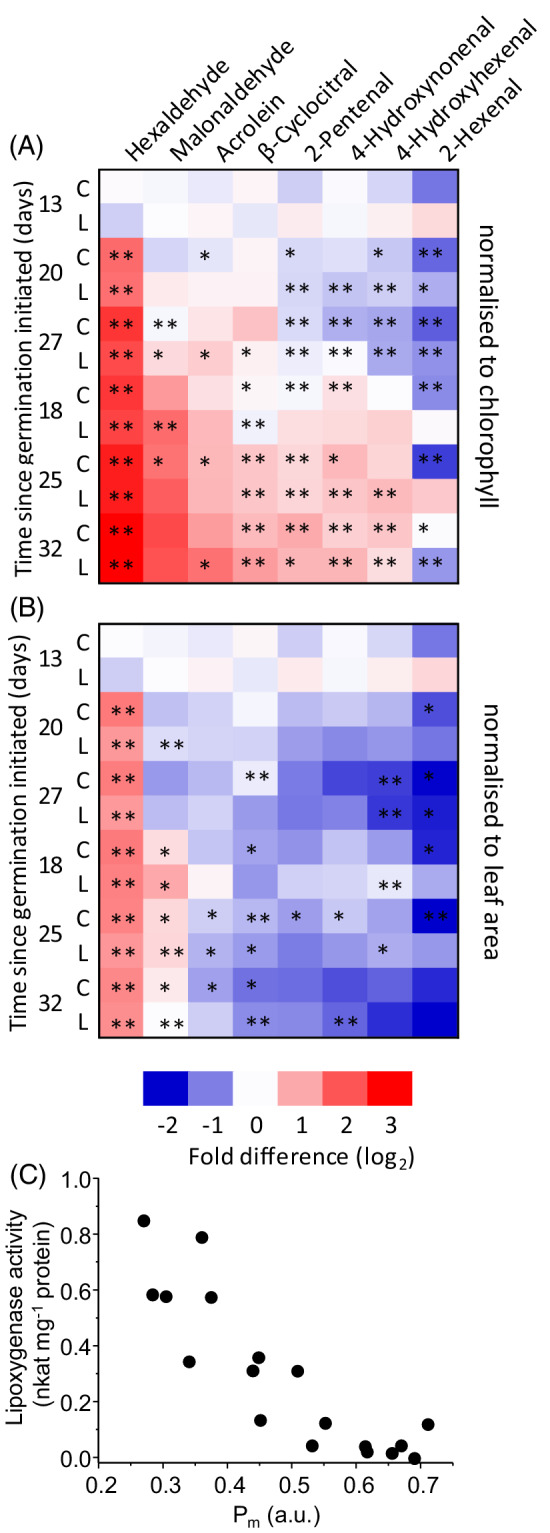
Concentrations of aldehydes and RES, as markers of lipid peroxidation, during sequential senescence of first‐emerging barley leaves. Differences for cv. Carina (C) and cv. Lomerit (L), and for each time after sowing seeds (indicated left), are shown relative to pre‐senescent leaves (average of both cultivars 13 days after sowing seeds), as log_2_ values on a colour scale (shown below). Data have been normalised to (A) total chlorophyll, and (B) measured leaf area, with * and ** denoting significant differences at *p* < 0.05 and *p* < 0.01, respectively, *n* = 6 leaves for each cultivar at each time point. (C) Leaf lipoxygenase activity in cv. Carina relative to maximum redox change of photosystem I (*P*_m_), used as a senescence marker

When normalised to total chlorophyll, concentrations of hexaldehyde and RES increased during senescence, except for 2‐hexenal (Figure 3A). Correlation of absolute concentrations with *P*_m_ generally revealed log‐normal relationships for all species quantified (Figure S5). Hexaldehyde was the only species that significantly increased consistently after 13 days since sowing seeds, and has the strongest log‐normal *R* ^2^ value with a *P*_m_ of 0.61 (Figure S5). This is partly due to low hexaldehyde concentrations (<0.5 μmol mol^−1^ total chlorophyll) only found in leaves with a *P*_m_  > 0.45 and high concentrations (>3.0 μmol mol^−1^ total chlorophyll) only found in leaves with a *P*_m_ < 0.3 (Figure S4). The RES acrolein was at a similar concentration to hexaldehyde (up to 4.5–5.5 μmol mol^−1^ total chlorophyll; Figure S5), but increased less during senescence (Figure 3A). Overall, relationships of RES with *P*_m_ were relatively weak. For example, malondialdehyde, often the most abundant species (up to 14 μmol mol^−1^ total chlorophyll), had a log‐normal *R* ^2^ value with *P*_m_ of 0.15 when considering both cultivars together. In pre‐ or early‐senescing leaves (*P*_m_ >0.5), concentrations of β‐cyclocitral were 0.2–0.3 μmol mol^−1^ total chlorophyll, and the ratio of β‐cyclocitral:β‐carotene are ca. 1:2 × 10^6^, while in late‐senescing leaves (*P*_m_ <0.2) β‐cyclocitral concentrations were up to 0.9 μmol mol^−1^ total chlorophyll and have a typical ratio to β‐carotene of 1:1 × 10^5^. However, some late‐senescing leaves possessed equal β‐cyclocitral concentrations as pre‐senescent leaves, leading to a low log‐normal *R* ^2^ value with *P*_m_ of 0.38 (Figure S5). Similar to formaldehyde (Figure S4), other short‐chain aldehydes, including acetylaldehyde, propionaldehyde, butyraldehyde, benzylaldehyde and valeraldehyde only significantly increased, relative to chlorophyll, at late stages of senescence (data not shown).

When normalised to leaf area, hexaldehyde was the only species consistently found at higher concentrations at 18–32 days, compared to 13 days, after sowing seeds (Figure 3B). Lipoxygenase activity, a potential source of hexaldehyde, was barely detectable in leaves before senescence and only increased when *P*_m_ decreased to values <0.5, after which activity increased as senescence progressed (Figure 3C). On a leaf area basis, the RES acrolein, 4‐hydoxynonenal (HNE), 4‐hydroxyhexenal (HHE) 2‐pentenal and 2‐hexenal declined as senescence progressed (Figure 3B), concomitantly with the decrease in chlorophyll content. No senescence‐related increase in light‐induced O_2_^•−^/H_2_O_2_ production, as measured with EPR spin‐trapping, in leaves of either genotype was observed during the observation period (Figure S6).

Together, normalisation to chlorophyll and leaf area indicate that RES are generated within the chloroplast, that the production is probably linked to ^1^O_2_ production rather than O_2_^•−^/H_2_O_2_ production, but that these RES do not accumulate in leaves early in senescence.

### 3.3 α‐Tocopherol and lipid composition

**FIGURE 4:**
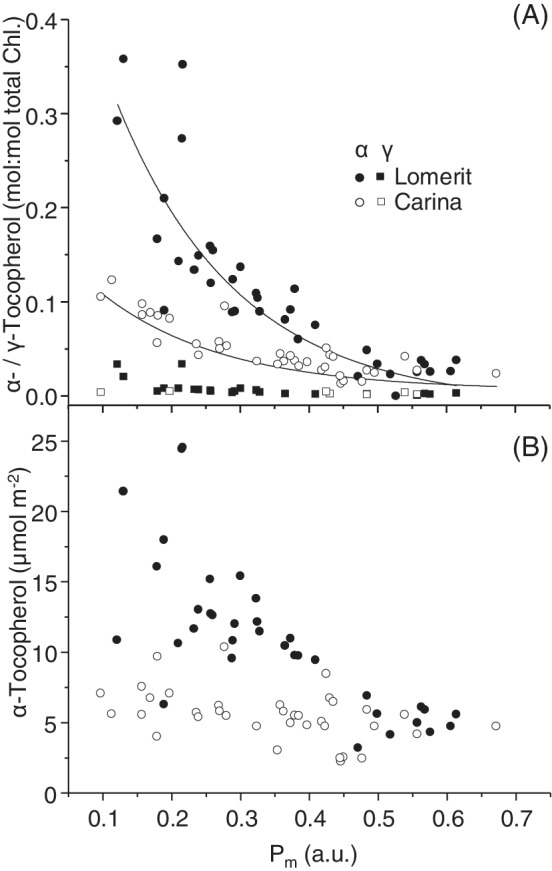
Relationship between maximum redox change of photosystem I (*P*_m_), used as a senescence marker, and tocopherol concentrations during sequential senescence in first‐emerging barley leaves. (A) α‐tocopherol (circles) and γ‐tocopherol (squares) normalised to total chlorophyll in leaves of cv. Lomerit (closed symbols) and cv. Carina (open symbols). Each data point represents an individual leaf with all data shown from both time courses. (B) Concentrations of α‐tocopherol normalised to leaf area in cv. Carina and cv. Lomerit

Concentrations of α‐tocopherol, relative to total chlorophyll, consistently increased during senescence (Figure 4A), as shown by log‐normal *R* ^2^ values with *P*_m_ of 0.75 and 0.81 for cv. Lomerit and cv. Carina, respectively. γ‐tocopherol changed similarly, but was at lower concentrations than α‐tocopherol, with half the samples below the detection limit (Figure 4A). A similar trend was observed when using chlorophyll content as a senescence marker (Figure S4). When normalised to leaf area, the same trend of increased concentrations as senescence progressed is apparent (Figure 4B), although with weaker *R* ^2^ values of 0.54 and 0.13, respectively. In both cases, concentrations in cv. Lomerit increased more than cv. Carina, and the relationship with chlorophyll was closer than with leaf area (Figure 4).

**FIGURE 5:**
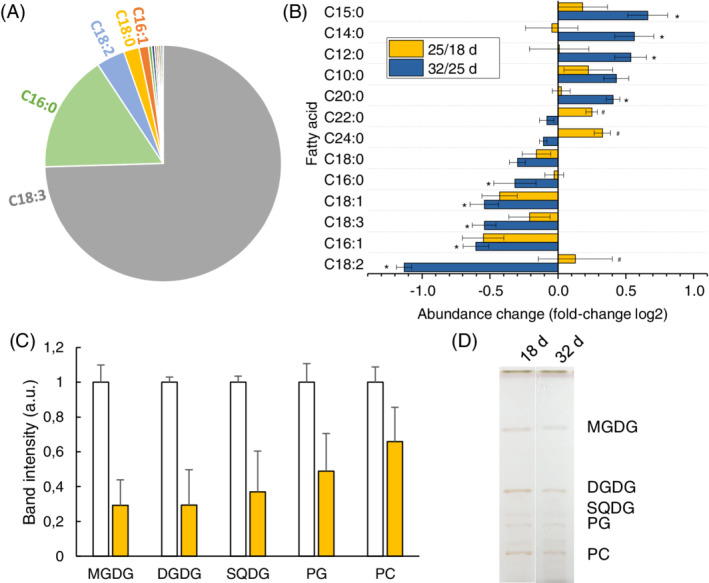
Changes in fatty acids (FA) and membrane lipids during sequential senescence in first‐emerging barley leaves of cv. Carina. (A) Percentage of each FA in total leaf FAs, in pre‐senescent leaves 18 days (d) after sowing seeds. Species are denoted by acyl chain length followed by number of double bonds; linolenic acid (C18:3), palmitic acid (C16:0), linoleic acid (C18:2), stearic acid (C18:0) and palmitoleic acid (C16:1). (B) Fold difference of each fatty acid between 18 d and 25 d (blue), and 25 d and 32 d (yellow) on a log_2_ scale. Asterisks denote a significant change compared to the previous time period (*p* < 0.05). (C) Relative difference of membrane lipids between 18 d and 32 d (*n* = 4), as quantified by band intensity after separation of total lipid extracts by thin layer chromatography, as shown in (D). All data are normalised to leaf DW.

Lipid composition during senescence was monitored by changes in amounts of each fatty acid and membrane group in cv. Carina. Eighteen days after sowing seeds, linolenic acid (C18:3) constituted 75% of the total fatty acid pool in the first leaves, followed by palmitic acid (C16:0; 16%), linoleic acid (C18:2; 4%), stearic acid (C18:0; 2%) and palmitoleic acid (C16:1; 1%), with unsaturated fatty acids composing 80% of total fatty acids (Figure 5A). After 25 days since sowing (mid senescence, *P*_m_ had decreased by 40%–45%) levels of unsaturated fatty acids decreased, while levels of saturated fatty acids increased, especially short‐chain (C10:0–C15:0) species, with the exception of C16:0 that decreased (Figure 5B). This trend tended to continue to 32 days (late senescence, *P*_m_ decreased by ca. 65%), but to a lesser extent (Figure 5B), so that unsaturated fatty acids composed 72% of total fatty acids. Concerning membrane groups, galactolipids (MGDG, DGDG and SQDG) of the thylakoid membrane were most abundant in the first leaves 18 days after onset of germination (Figure 5C). They decreased to a greater extent than the detected phospholipids (PG, PC) (Figure 5D). This clearly demonstrates that thylakoid membranes are degraded during senescence.

### 3.4 Principal component integration of photosynthetic and lipid‐associated parameters

**FIGURE 6:**
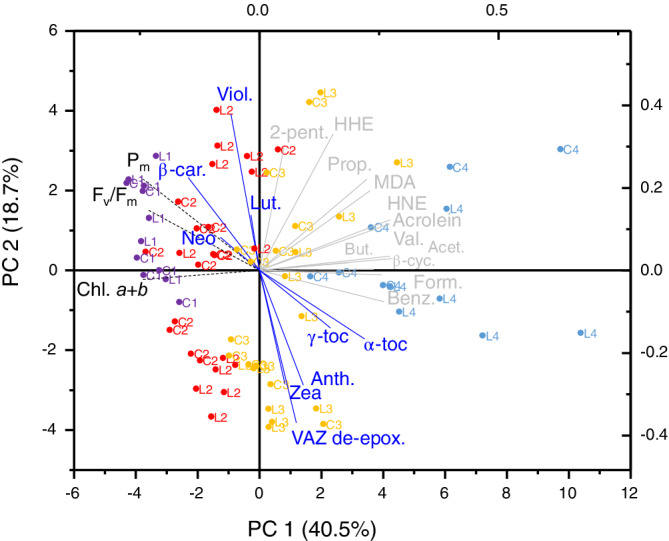
A bi‐plot principal component analysis (PCA) of photosynthetic and lipid peroxide‐related parameters during developmental senescence of first‐emerging barley leaves. Each data point, separated in PC1 (bottom: x‐axis) and PC2 (left: y‐axis), represents an individual mid‐leaf segment of cv. Lomerit (L) and cv. Carina (C), coloured by age, into Groups 1 (13 days since sowing; purple), 2 (18 or 20 days since sowing; red), 3 (25 or 27 days since sowing; yellow) or 4 (32 days since sowing; green). Overlaid is the loading plot (right: *y*‐axis; top: *x*‐axis), showing established senescence indicators (black dotted line), photosynthetic pigments and tocopherols (blue), and all detected lipid peroxide breakdown products (grey).

Principal component (PC) analysis of all aforementioned data measured in both cultivars, and using relative changes of aldehyde/RES concentrations normalised to chlorophyll, separated samples due to age on PC1, which accounted for 40.5% of the total variance (Figure 6). Of note, the grouping amongst leaves of the same age on PC1 weakened as leaves aged, as did the overlap between groups of leaves in each age category, highlighting increasing variability between leaves as senescence progressed. The loading plot showed that decreases in established senescent markers (i.e. *F*_v_/*F*_m_, *P*_m_, total chlorophyll) separated leaves along PC1 in a different direction to the increase in tocopherols and aldehydes and RES, with the exception of RES trans‐2‐pentenal and HHE since these two species showed only a weaker relationship with leaf age. Variation between leaves in every age category separated on PC2, which accounted for 18.7% of the total variance, and was influenced by differences in carotenoids between leaves of the same age (Figure 6).

## 4 DISCUSSION

The production of ROS is unavoidable from excited chlorophyll and during photosynthetic electron transport, which can lead to lipid peroxidation of PUFA‐rich thylakoid membranes and the production of lipid hydroperoxides. Aldehydes and RES are subsequently released from spontaneous or ROS‐dependent decomposition and hydroperoxide lipase activity (Farmer & Mueller, 2013; Griffiths et al., 2000). Various pathways exist for further breakdown, because the accumulation of such species leads to cell toxicity (Farmer & Mueller, 2013; Mano, 2012; Roach et al., 2018). Although stress‐induced senescence is associated with lipid peroxidation, and studies with antioxidant knock‐out mutants have shown that elevated RES levels can induce senescence (Nurbekova et al., 2021; Srivastava et al., 2017), it remains open if ROS‐induced RES production plays a role in non‐stress associated developmental senescence.

In the two barley varieties investigated here, several RES accumulated during senescence when RES was normalised to chlorophyll (Figure 3A). These included acrolein, β‐cyclocitral, 4‐HHE and HNE that are known to be generated by ROS. Inactivation of the photosynthetic electron transport chain leads to excitation pressure in PSII and consequently an increase in ROS production. During senescence, photosynthetic electron transport decreased first at the level of PSI, as seen by the loss in *P*_m_, followed by a loss in *F*_v_/*F*_m_, reflecting a loss in active PSII (Figure 1), potentially leading to elevated ROS production. ^1^O_2_ is a major source of lipid peroxidation in the chloroplast, but ^•^OH radicals derived from O_2_^•−^/H_2_O_2_ are also able to oxidise lipids. In both first and flag leaves, ^1^O_2_ production increased only in cv. Lomerit during senescence when measured in isolated thylakoids (Krieger‐Liszkay et al., 2015; Shimakawa et al., 2020). In the presence of α‐linolenic acid, ^1^O_2_ can lead to the formation of acrolein, which accumulates under stress in the chloroplast (Mano, 2012; Roach et al., 2020). The increase in acrolein was significant for cv. Lomerit, but not for cv. Carina at the latest stage of senescence (Figure 3A), and a significant effect of cultivar was found over all time points (Table S1). Other non‐enzymatic lipid peroxide products of α‐linolenic acid include HHE and malondialdehyde, while HNE is produced from linoleic acid (Schneider et al., 2005), but no clear pattern connected these RES during senescence. In addition, β‐cyclocitral, generated by ^1^O_2_‐induced oxidation of β‐carotene, and potentially involved in ^1^O_2_ signalling (Ramel et al., 2013), increased at late senescent stages relative to chlorophyll, but no clear difference between cv. Lomerit and cv. Carina was observed. During senescence, O_2_^•−^ levels increased in thylakoids isolated from flag leaves of the two barley cultivars grown in the field (Krieger‐Liszkay et al., 2015), but not in first leaves (Figure S6; Shimakawa et al., 2020). This difference may be caused by a higher antioxidant level in chloroplasts in first leaves than in flag leaves. A gradual increase in lipid peroxidation and a transient increase in O_2_^•−^/H_2_O_2_ were observed during senescence in a study on second barley leaves, with the highest levels of lipid peroxidation occurring at the late stages of senescence (Jajić et al., 2015).

In contrast to the increase in RES and aldehyde contents during senescence relative to total chlorophyll, hexaldehyde was the only species that consistently accumulated relative to leaf area (Figure 3B). Previously, a chloroplast‐located lipoxygenase, 13‐LOX that binds to the chloroplast membrane of barley leaves, was identified to break down this plastid structure during senescence to release stromal constituents (Springer et al., 2016). 13‐LOX catalyses stereospecific oxygenation of fatty acids forming 13‐hydroperoxides, which are further broken down by 13‐hydroperoxide lyase to release aldehydes. When linoleic acid is the substrate of 13‐LOX, hexaldehyde is the product of the lyase, whereas with linolenic acid hexenal is produced (Montillet et al., 2004). Therefore, the accumulation of hexaldehyde and the decrease in linoleic acid support an involvement of 13‐LOX‐mediated degradation of the chloroplast membrane during senescence. In support of this, lipoxygenase activity increased as senescence progressed (Figure 3C). The LOX pathway may also have a role in senescence via the synthesis of jasmonates (Hu et al., 2017; Seltmann et al., 2010), which in monocot plants (i.e. barley) further promote senescence (Bachmann et al., 2002).

As shown in Figure 3 and Figure S4, there is no clear difference in the concentrations of most RES between the two barley cultivars despite that cv. Lomerit produces more ^1^O_2_ (Shimakawa et al., 2020). This suggests that the antioxidant system is capable of controlling RES levels in both cultivars. Zeaxanthin and α‐tocopherol levels increased during senescence in both cultivars but considerably more for cv. Lomerit (Figure 4; Table S1). Since α‐tocopherol can very efficiently scavenge ^1^O_2_ (Krieger‐Liszkay & Trebst, 2006), these results indicate, first, that barley leaves were preventing ROS‐mediated lipid peroxidation by elevating α‐tocopherol concentrations, and second, why ROS‐associated RES levels were generally similar between cultivars. However, the increase of α‐tocopherol may also reflect an increase in size and number of plastoglobuli, a phenomenon commonly observed during leaf senescence (van Wijk & Kessler, 2017). It should also be noted that RES are scavenged in leaves by enzymes, such as various aldo‐keto reductase/dehydrogenases/oxidases and 2‐alkenal reductase (Mano, 2012; Shimakawa et al., 2014). In *A. thaliana* leaves, elevating aldehyde dehydrogenase levels accelerated aldehyde breakdown and improved stress tolerance (Sunkar et al., 2003), while in leaves and siliques, aldehyde oxidases break down natural levels of RES under growth chamber conditions, preventing premature senescence (Nurbekova et al., 2021; Srivastava et al., 2017). Production of ^1^O_2_ can also be prevented in the first place by thermal dissipation of excess light energy (i.e. NPQ) (Roach & Krieger‐Liszkay, 2019). Therefore, the increase in NPQ during senescence (Figure 2) may also have helped to prevent ^1^O_2_‐mediated lipid peroxidation. NPQ levels were a bit lower in cv. Carina than in cv. Lomerit, and similar to the difference in VAZ de‐epoxidation levels, there was a significant effect of cultivar for antheraxanthin and zeaxanthin when considering all time points collectively (Table S1). According to the PCA, variation in xanthophyll composition was a cause for the separation of leaves of the same age of both cultivars, indicating that each leaf had experienced different light exposure at the time of harvesting. Therefore, adjustments to NPQ involving antheraxanthin and zeaxanthin were likely compensating for short‐term changes in light exposure, helping prevent ROS production. An increase in NPQ accompanied by an increase in zeaxanthin and antheraxanthin during senescence has been observed previously in field‐grown wheat leaves (Lu et al., 2001).

In summary, the first step of senescence in the greenhouse‐grown barley cultivars was the decrease in chlorophyll contents and *P*_m_ values, followed later by a loss of *F*_*v*_/*F*_*m*_ and an increase in NPQ (Figures 1 and 2). Lipoxygenase activity increased, and 13‐LOX activity using linoleic acid as substrate was detected via accumulation of hexaldehyde. β‐carotene seems to decrease linearly with PSI activity (Figure S3), while α‐tocopherol started to increase later in an exponential manner (Figure 4). In *A. thaliana* upon light stress, first α‐tocopherol is consumed and then, in a second stage, carotenoids (Kumar et al., 2020). However, in the first barely leaves, our data support the hypothesis that ^1^O_2_ reacts first with β‐carotene in the reaction centres and, when this is largely consumed, α‐tocopherol acts as a scavenger. This shows that the importance of the antioxidants may differ depending on the physiological situation. Since β‐carotene oxidation products have signalling roles, it would be tempting to suggest that they act as a trigger of the signalling pathway leading to controlled natural senescence. However, β‐cyclocitral, like other ^1^O_2_‐derived RES, did not increase early during senescence, suggesting that such species likely do not trigger the onset of natural developmental senescence in barley, although they may be involved in signalling events triggering later steps of senescence.

## AUTHOR CONTRIBUTIONS

Ginga Shimakawa and Anja Krieger‐Liszkay conceived the study. Ginga Shimakawa and Thomas Roach performed the experiments. Ginga Shimakawa prepared the data. Thomas Roach prepared the figures. Thomas Roach and Anja Krieger‐Liszkay wrote the manuscript. All authors proof‐read the final submission.

## Supporting information


**Data S1:** Supporting Information

## Data Availability

The data that support the findings of this study are available from the corresponding author upon reasonable request.
