# Supplementary material for: ROS‐derived lipid peroxidation is prevented in barley leaves during senescence
Source: Physiol Plant. 2022 Sep 12;174(5):e13769. doi: 10.1111/ppl.13769 (PMC9544269; doi:10.1111/ppl.13769)
Supplement: Supplementary file 1 — Data S1: Supporting Information [file PPL-174-0-s001.pdf]

Supplemental Table 1. Statistical analysis using a univariate general linear model to calculate P values between the cultivars and over time, including data from both time courses (n = six replicates each for the six time points). Significant values where P<0.05 are labelled red.

	P values			
	**Cultivar**	**Time**	**Cultivar**	**Time**
**F_v/F_m**	0,699	<0.001		
**P_m**	0,479	<0.001		
	**Normalised to chlorophyll**		**Normalised to leaf area**	
**Total Chlorophyll**			0,238	<0.001
**Chl *a*:*b***			0,824	0,005
**VAZ DEP (A+Z)/(V+A+Z)**			0,169	<0.001
**Acrolein**	0,007	<0.001	0,001	<0.001
**Propionaldehyde**	0,506	<0.001	0,475	<0.001
**Malondialdehyde**	0,634	<0.001	0,084	<0.001
**trans-2-hex**	0,011	<0.001	0,006	<0.001
**trans-2-pent**	0,176	0,061	0,111	<0.001
**β-cyclocitral**	0,956	<0.001	0,007	<0.001
**HHE**	0,545	0,072	0,155	<0.001
**HNE**	0,989	<0.001	0,68	<0.001
**Formaldehyde**	0,118	<0.001	0,95	0,002
**Acetylaldehyde**	0,056	<0.001	0,369	0,005
**Hexaldehyde**	0,56	<0.001	0,003	<0.001
**Butyraldehyde**	<0.001	<0.001	<0.001	<0.001
**Benzylaldehyde**	0,953	<0.001	0,078	<0.001
**Valeraldehyde**	0,001	<0.001	<0.001	0,001
**Neoxanthin**	0,065	0,133	0,32	0,014
**Violaxanthin**	0,222	0,002	0,364	0,435
**Antheraxanthin**	<0.001	0,082	0,897	<0.001
**Lutein**	0,145	0,812	0,541	0,12
**Zeaxanthin**	0,024	0,129	0,618	0,028
**β-carotene**	0,113	<0.001	0,27	<0.001
**α-tocopherol**	<0.001	<0.001	0,13	<0.001

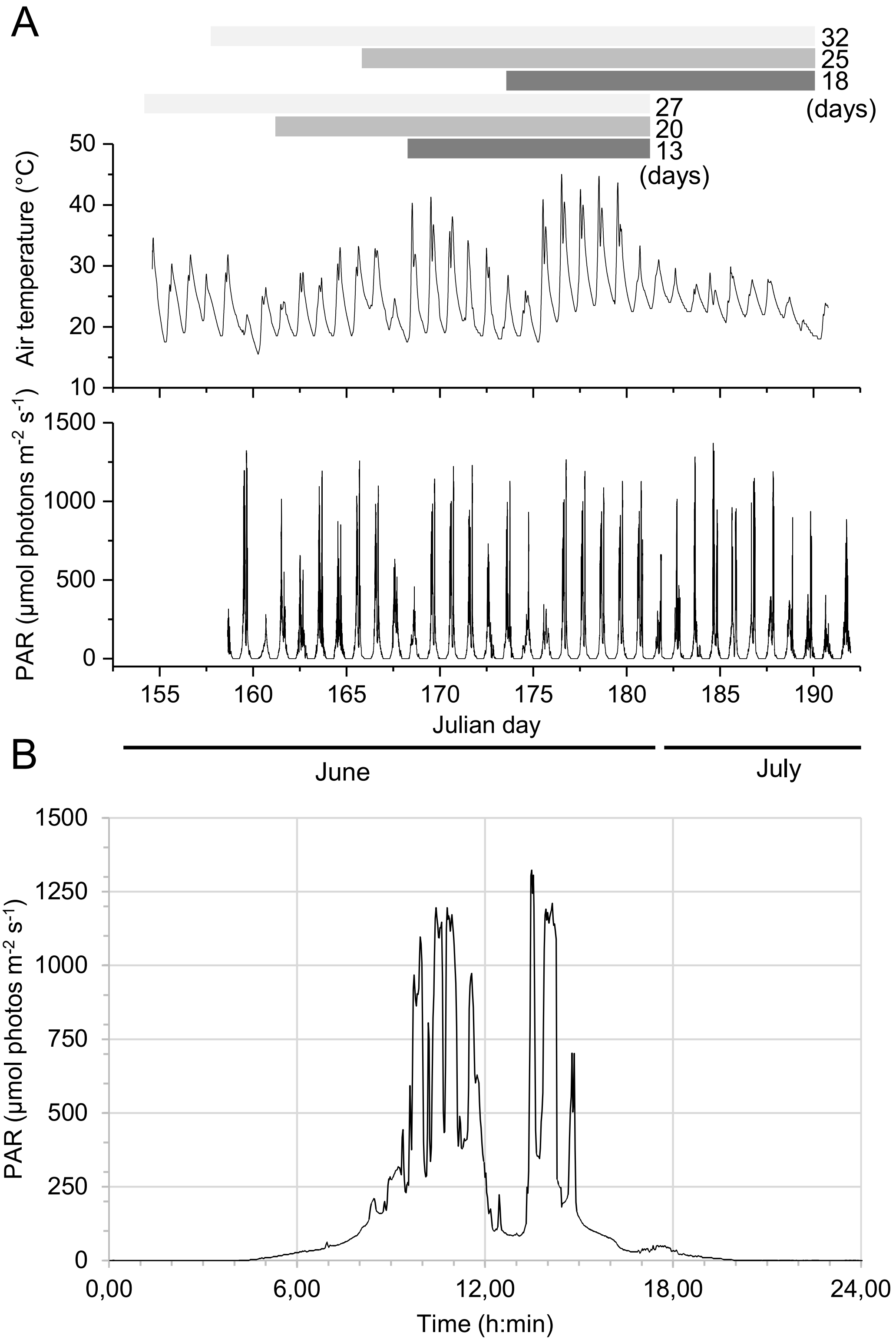

Supplementary Figure 1. Climatic conditions in the glass house used for growing the barley plants. (A) The grey bars above indicate when plants were grown, starting with the day when seeds were sown and ending when measurements were made. (B) Typical light intensity on a cloudless day. PAR: Photoysnthetic active radiation

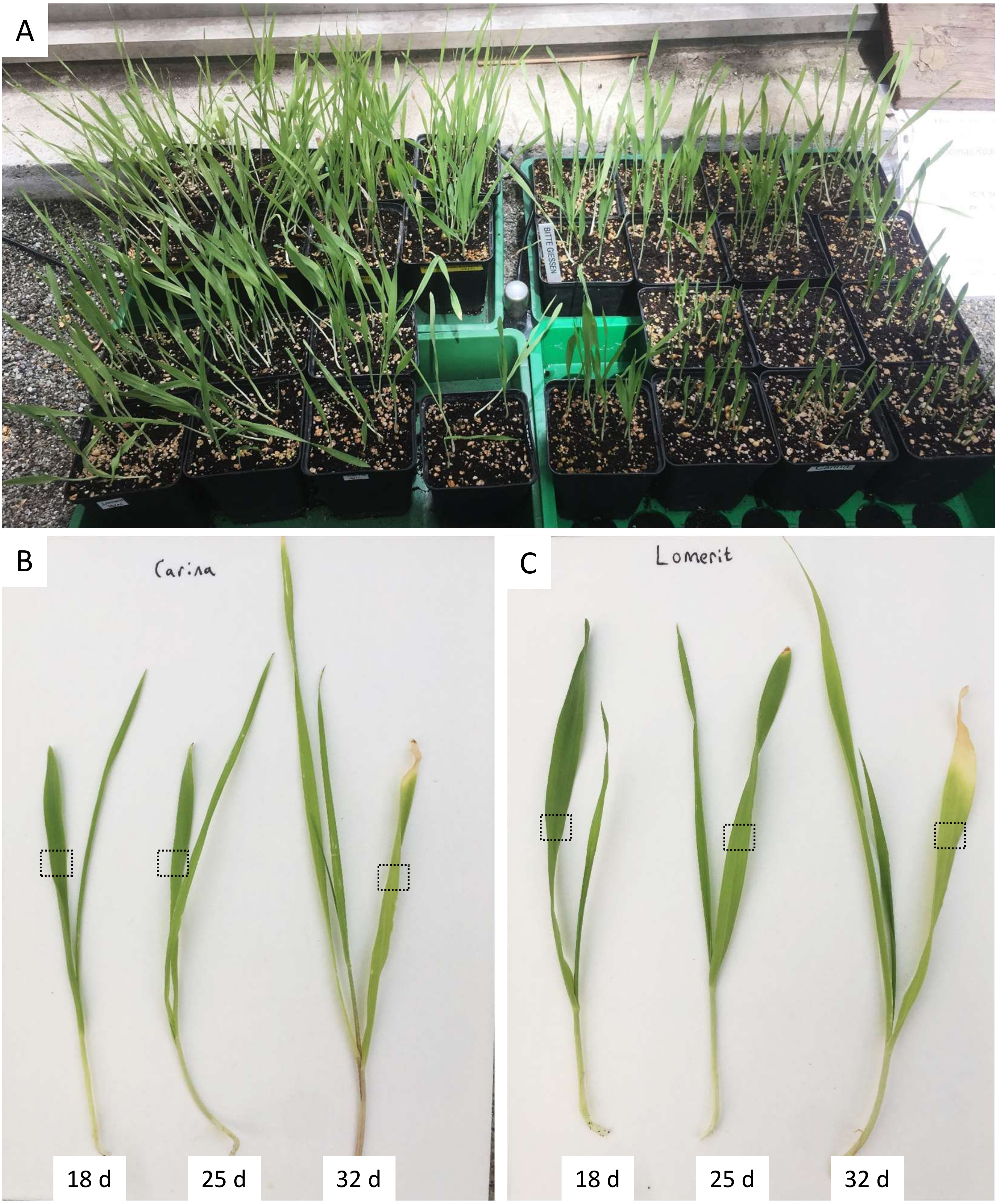

Supplementary Figure 2. Image of the barely plants of different ages used for experiments, here just after watering. Typical plants after 18, 25 and 32 days (d) since sowing of (B) cv. Carina and (C) cv. Lomerit. The black dashed boxes indicate the midpoint of first leaves measured.

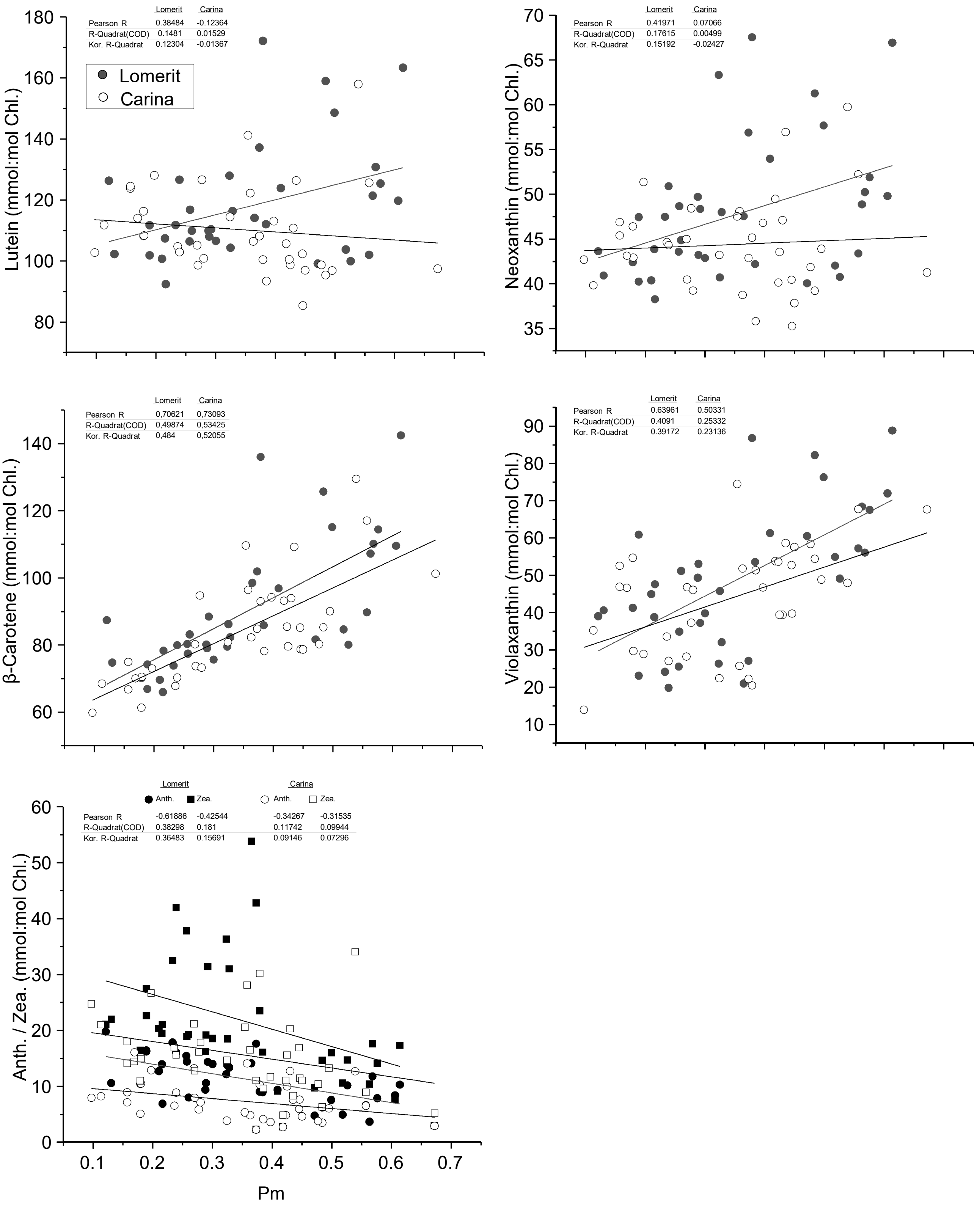

Supplementary Figure 3. Concentrations of carotenoids, normalised to total chlorophyll content (Chl.), relative to changes in maximum photo-oxidizable P700 levels (P_m) in first leaves of barley cultivars Lomerit and Carina.

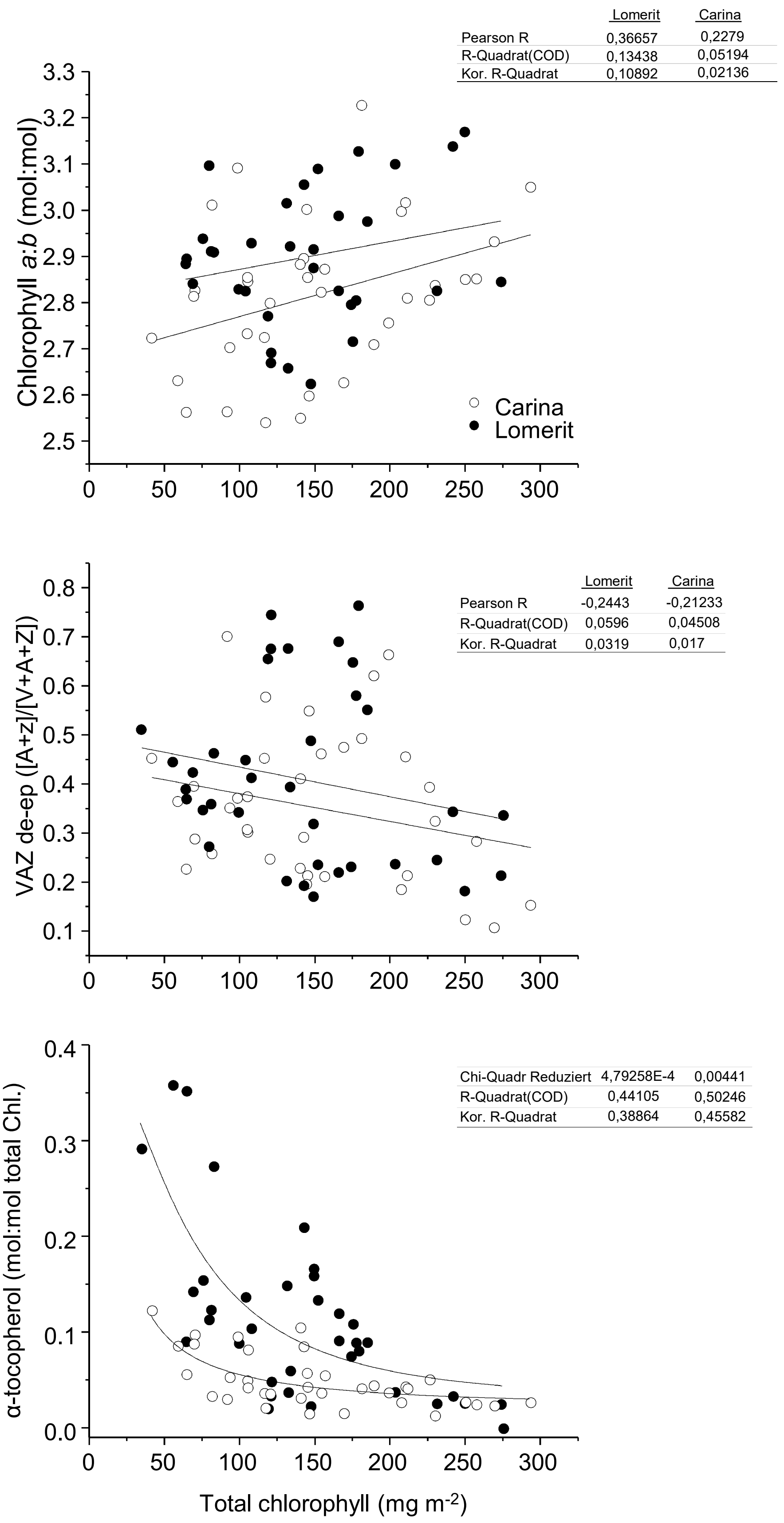

Supplementary Figure 4. Chlorophyll *a*:*b* ratio, xanthophyll cycle de-epoxidation (de-ep) ratio and tocopherol concentrations, relative to changes in total chlorophyll (Chl.) levels, in first leaves of barley cultivars Lomerit (filled symbols) and Carina (open symbols). A: antherxanthin, Z: zeaxanthin, V: violxanthin.

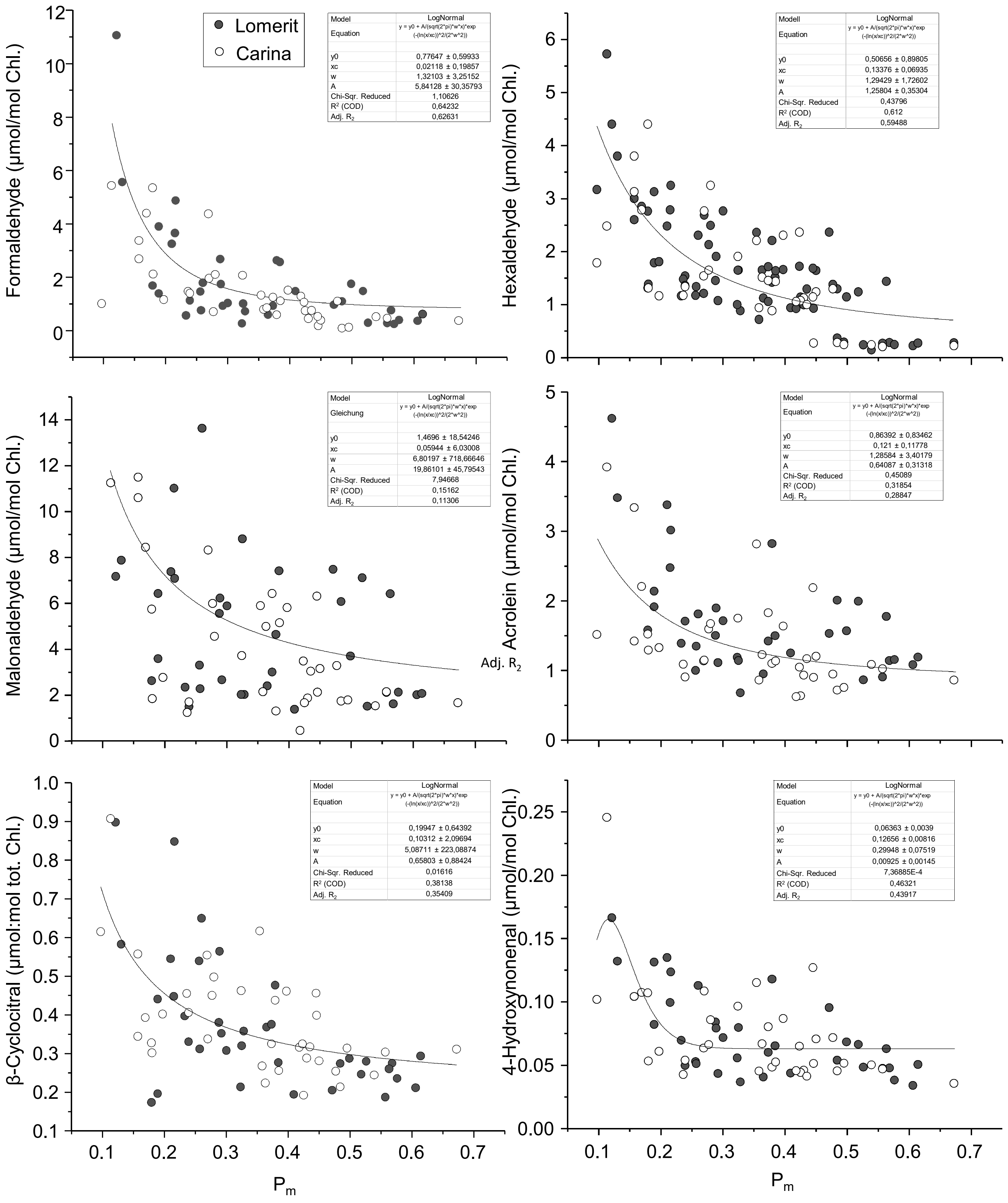

Supplementary Figure 5. Absolute concentrations of aldehyde, including reactive electrophile species (malonaldehyde, acrolein, β-cyclocitral and 4-Hydroxynonenal), normalised to total chlorophyll content (Chl.), relative to changes in maximum photo-oxidizable P700 levels (P_m) in first leaves of barley cultivars Lomerit (filled symbols) and Carina (open symbols).

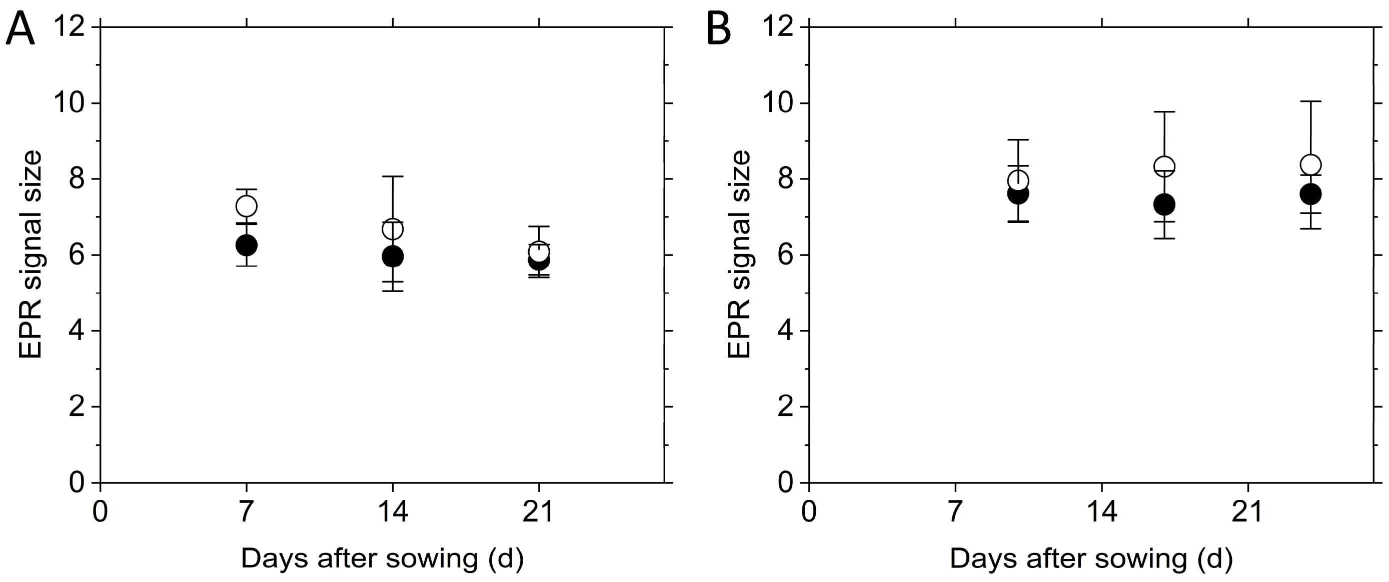

Supplementary Figure 6. $O_2^{\bullet -}/H_2O_2$-derived hydroxyl radical production in leaves measured by spin trapping EPR spectroscopy Size of the of 4-POBN/a-hydroxyethyl adduct in illuminated leaves of barley, cv Lomerit (closed circles) and cv. Carina (open circles). The experiments were performed independently in two series: 17, 14, and 21 days after sowing (A); and 10, 17, and 24 days after sowing (B). The EPR signal sizes were normalized to chlorophyll content of each sample. Data are shown as the mean with standard deviation of three biological replicates.
